# 3D-QSAR Study of Combretastatin A-4 Analogs Based on Molecular Docking

**DOI:** 10.3390/molecules16086684

**Published:** 2011-08-08

**Authors:** Yinghua Jin, Ping Qi, Zhiwei Wang, Qirong Shen, Jian Wang, Weige Zhang, Hongrui Song

**Affiliations:** 1 Department of Pharmacy, General Hospital of Beijing Military Command. Nanmencang No.5, Dongcheng District, Beijing 100700, China; 2 School of Pharmaceutical Engineering, Shenyang Pharmaceutical University. No.103, Wenhua Road, Shenhe District, Shenyang 110016, Liaoning, China

**Keywords:** CA-4, antitumoral and antivascular activities, dock, CoMFA

## Abstract

Combretastatin A-4 (CA-4), its analogues and their excellent antitumoral and antivascular activities, have attracted considerable interest of medicinal chemists. In this article, a docking simulation was used to identify molecules having the same binding mode as the lead compound, and 3D-QSAR models had been built by using CoMFA based on docking. As a result, these studies indicated that the QSAR models were statistically significant with high predictabilities (CoMFA model, q^2^ = 0.786, r^2^ = 0.988). Our models may offer help to better comprehend the structure-activity relationships for this class of compounds and also facilitate the design of novel inhibitors with good chemical diversity.

## 1. Introduction

**Figure 1:**
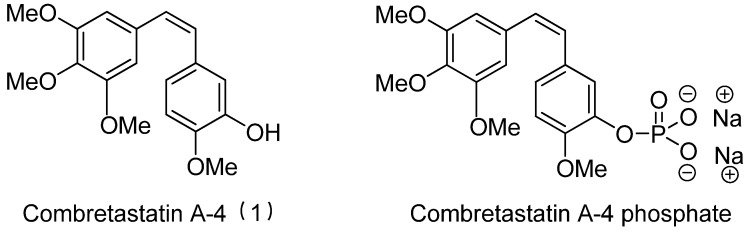
Combretastatin A-4 and its water-soluble sodium phosphate prodrug CA-4P.

Combretastatin A-4 (CA-4) (Figure 1) was isolated from the bark of the South African tree *Combretum caffrum* in 1989 by Pettit and co-workers [1]. This *cis*-stilbene strongly inhibits tubulin polymerization by binding to the colchicine site and is found to be a cytotoxic agent against a wide variety of tumor cell lines, including multidrug-resistant lines [2,3]. The water-soluble sodium phosphate prodrug CA-4P (Zybrestat ^TM^) (Figure 1) is currently in several advanced clinical trials for age-related macular degeneration and anaplastic thyroid cancer based on the vascular shutdown mechanism of action [4,5].

Due to its excellent antitumor and antivascular activities, CA-4 has attracted considerable interest of medicinal chemists in the design and preparation of analogs as novel antitumor agents. In the past 20 years, thousands of its analogs have been synthesized, and some CoMFA studies have been published [6,7]. These studies on the CA-4 analogues have revealed some detailed and instructional information on the effects of structural modification at several substitution positions. However, molecules used in previous CoMFA studies lacked molecular diversity because of their similar design ideas. Therefore, the obtained models might fail to predict other series of analogs.

For the purpose of achieving a more accurate CA-4 action model, CA-4 analogs were collected from different studies insamuch as possible. We envisaged that docking before CoMFA could help us identify which analogs have the same binding mode as CA-4. These selected molecules would then have chemical diversity, and a mode of action similar to that of CA-4.

When CoMFA is performed based on the docking alignment, we encountered a problem that the data from different sources are hard to compare and analyse. In other words, the values of CA-4 are not the same in different studies. The normalization method of processing data has been tested and the results prove its feasibility. Details are as follows: active value of CA-4 is set to 1, relative activity = IC_50_ (Compound)/IC_50_ (CA-4). When the compounds have activities higher than CA-4, the relative activities are less than 1, and *vice versa*. 

In this way, a kind of uniform comparability among the active value of different compounds from different studies has been built. At the same time, this normalization method provides a strong guarantee for our following 3D-QSAR study, and has laid a foundation for the design of new derivatives able to act as potent tubulin polymerization inhibitors.

## 2. Methods

### 2.1. Data Sets

The CA-4 analogues used for docking studies, with two-atom bridgeheads, were obtained from literature [8,9,10,11,12,13,14,15,16,17,18,19,20,21,22,23,24,25,26,27,28,29,30].

### 2.2. Molecular Docking

Molecular docking studies were performed with Molegro Virtual Docker 2008.3.0.0 to investigate possible binding modes for all studied compounds [31]. All parameters used in docking were the defaults. For example: the default radius of 10 was used. The grid resolution was 0.30 Å; the max iterations were 1,500; The max population size was 50 and the energy threshold was 100. 

### 2.3. Molecular Modeling and Alignment

All calculations were performed using SYBYL 6.91 on a Silicon Graphics Fuel workstation equipped with the IRIX 6.5 operating system [32]. Active conformation selection and molecular alignment are the most sensitive parameters to construct a more credible CoMFA model. However, at present, the crystal structure of this series of compounds has not been identified; therefore, docking alignment was used to construct the 3D-QSAR model.

### 2.4. CoMFA Descriptors

In this CoMFA study, the defaulting grid spacing of 2.0 Å was used. A variable column filtering energy cutoff was set at 2.0 kcal/mol to enhance the signal-to-noise ratio. CoMFA calculates steric fields using a Lennard-Jones 6–12 potential and electrostatic fields using a Coulombic potential, and an sp^3^ hybridized carbon atom probe with a van der Waals radius of 1.52 Å and a 1.0 charge was exploited in order to achieve the goal. Next, the model was optimized by setting steric and electrostatic field cutoffs at 30 kcal/mol. The steric and electrostatic ﬁelds were scaled making use of the CoMFA-STD method in SYBYL 6.91 [33,34]. The partial least-squares (PLS) methodology, which is an extension of multiple regression analysis, was applied to derive the 3D-QSAR, employing CoMFA descriptor as independent variables, and pIC_50_ values as dependent variables. To measure the predictive ability of the derived model, the cross-validations conducted through the leave-one-out procedure was carried out to obtain the optimal number of components. Then the ﬁnal non-cross-validated model was developed with an optimum number of components reported from the cross-validation results.

When the orientation of aligned molecules varied, the q^2^ value would change correspondingly. So the all-orientation search method (AOS) was executed by rotating the molecular aggregate within the grid to yield the highest q^2^ [35].

## 3. Results and Discussion

### 3.1. Validation of the Molecular Docking Reliability

CA-4 is a strong inhibitor of tubulin assembly by binding to the colchicine site, and in recent years, thousands of its analogues had been synthesized. There is no doubt that all of them have the same binding site as CA-4, yet these compounds exhibit different biological activities than CA-4. Therefore, we can reasonably assume that the binding capability to tubulin between them and CA-4 are not the same. In this study, we wished to identify the compounds which have the same binding modes as CA-4 through a docking study, followed by studies of their three-dimensional quantitative structure-activity relationships (3D-QSAR) to design a novel class of CA-4 mimics, capable of binding to the colchicine-binding site, displaying selective toxicity towards tumour vasculature and strongly inhibiting the tubulin polymerization.

**Figure 2:**
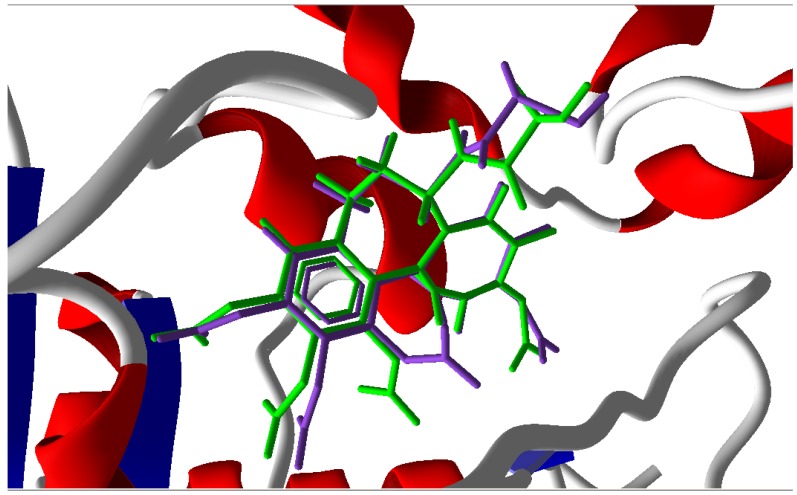
Binding conformations of the docked colchicine (purple) and crystal colchicines (green) at the active site of tubulin.

We collected compounds from the literature [8,9,10,11,12,13,14,15,16,17,18,19,20,21,22,23,24,25,26,27,28,29,30] as docking objects, the common characteristics of which were that they were all linked by two-atom chain. For the purpose of checking the accuracy of the docking study, the ligand colchicine was extracted from the crystal structure of complex (PDB ID: 1sa0) [36] and re-docked in the colchicine-binding site of tubulin [37]. As a result, we found that the docking conformation corresponding to the lowest MolDockScore was nearly identical to that found in the original X-ray structure, with an acceptable RMSD of 0.7 Å between the best scored conformation which obtained from docking and X-ray structure 1sa0 (Figure 2). Therefore, the Molegro Virtual Docker 2008.3.0.0 could be used for the following study.

### 3.2. Molecular Docking Results

**Figure 3:**
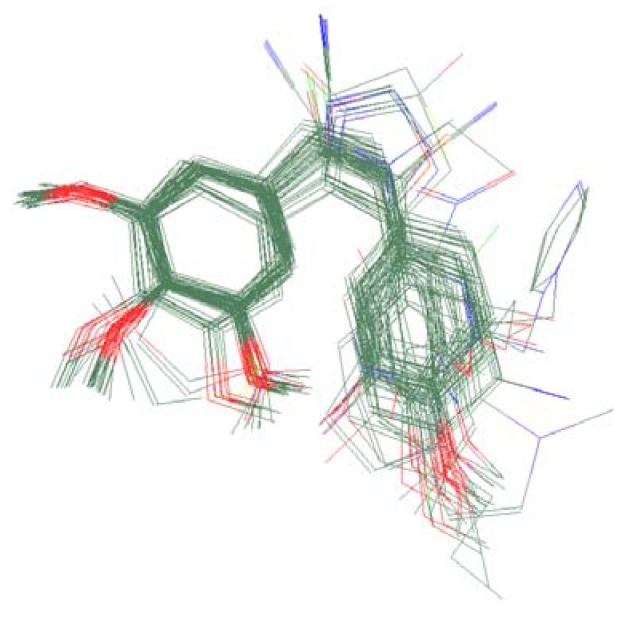
Alignment of all the studied compounds.

Since Molegro Virtual Docker can perfectly reproduce the reference binding modes observed in a crystal structure, it was used to dock all the ligands into the colchicine-binding site using the default parameters. The results of the docking study and visual analysis of the interactions suggested that among all studied compounds, there were 44 ligands whose binding modes were identical to that of CA-4. The structures of them were listed in Table 1. In the next step, we analyzed the docking scores for the 45 ligands. The lowest MolDockScore was selected and the values of them were listed in Table 2. However, only a low correlation was observed between docking scores and active values. This phenomenon, called false positive, is inevitable in any docking study [38]. The aligned compounds are shown in Figure 3.

**Table 1:** Structures having the same binding modes as CA-4 and used in the training and test set. 
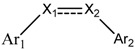

Compounds	Ar_1_	X_1_	X_2_	Ar_2_	Literature
1	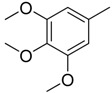	CH	CH	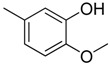	[8]
2	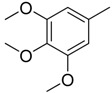	CO	CO	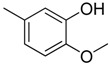	[9]
3	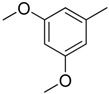	CH_2_	NH	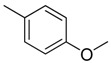	[10]
4	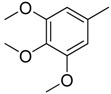	CH	CH	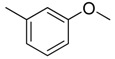	[10]
5	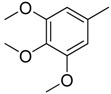	CH	CH	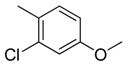	[10]
6	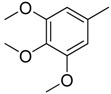	CH_2_	NCH_3_	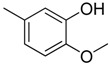	[11]
7	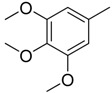	CH_2_	NCH_2_CH_3_	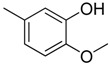	[11]
8	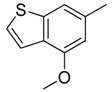	CH	CH	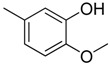	[12]
9	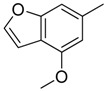	CH	CH	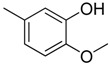	[12]
10	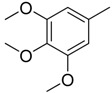	CF	CH	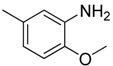	[13]
11	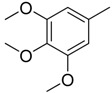	CF	CF	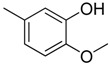	[13]
12	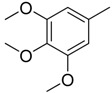	CF	CH	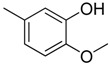	[13]
13	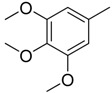	CH_2_	CH_2_	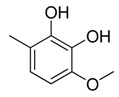	[14]
14	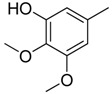	CH	CH	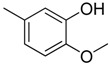	[14]
15	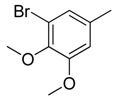	CH	CH	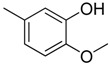	[15]
16	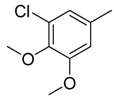	CH	CH	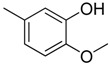	[15]
17	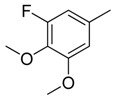	CH	CH	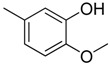	[15]
18	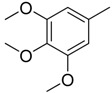	CH	CH	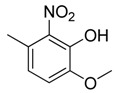	[16]
19	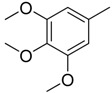	CCN	CH	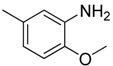	[17]
20	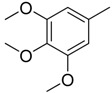	CH	CCN	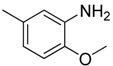	[17]
21	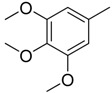	CCN	CH	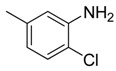	[17]
22	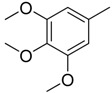	CH	CH	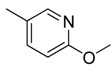	[18]
23	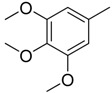	CH	CH	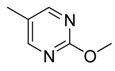	[18]
24	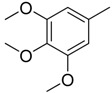	CH	CH	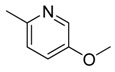	[18]
25	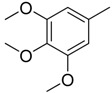	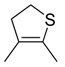		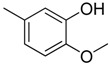	[19]
26	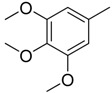	CH	CH	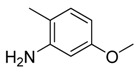	[20]
27	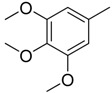	CH	CH	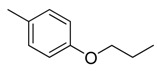	[22]
28	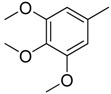	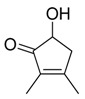		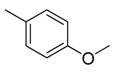	[21]
29	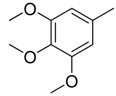	CH	CH	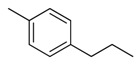	[22]
30	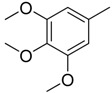	CHCN	CH_2_	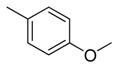	[22]
31	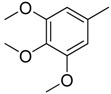	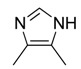		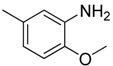	[23]
32	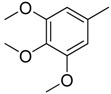	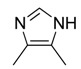		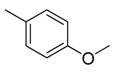	[23]
33	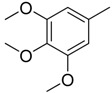	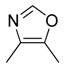		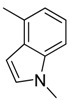	[23]
34	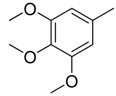	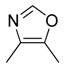		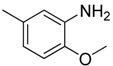	[23]
35	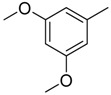	CH	CH	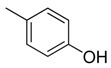	[24]
36	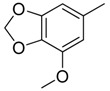	CH	CH	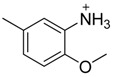	[25]
37	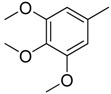	CH	CH	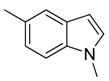	[26]
38	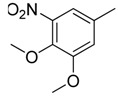	CH	CH	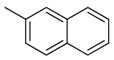	[26]
39	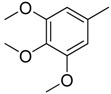	CH	CH	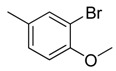	[27]
40	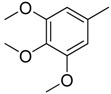	CH	CH	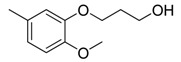	[27]
41	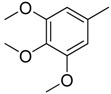	CH	CH	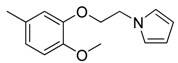	[27]
42	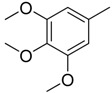	CH	CH	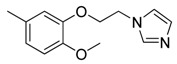	[27]
43	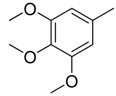	CH	CH	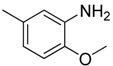	[28]
44	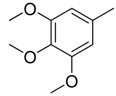	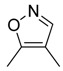		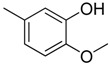	[29]
45	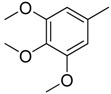	CH	CH	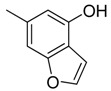	[30]

**Table 2:** Relative activities, predicted activities and docking results for all studied compounds.

Compound	Relative	Predicted	Residue	MolDockScore
1	0.00	0.03	−0.03	−92.11
2 ^t^	−0.18	−0.08	−0.10	−102.1
3	−1.18	−1.20	0.01	−83.85
4	−0.67	−0.68	0.01	−86.39
5	−0.26	−0.26	−0.01	−84.69
6	−1.06	−1.02	−0.04	−81.16
7	−0.59	−0.56	−0.02	−95.79
8	0.23	0.18	0.05	−84.34
9	−0.02	−0.03	0.01	−80.87
10	−0.05	−0.02	−0.03	−117.5
11	−0.17	−0.13	−0.04	−105.8
12	0.29	0.21	0.08	−103.8
13^ t^	−0.26	−0.21	−0.05	−88.41
14^ t^	-0.26	−0.06	−0.19	−90.39
15	0.08	0.10	−0.01	−85.25
16^ t^	0.05	−0.12	0.17	−82.73
17^ t^	0.08	−0.07	0.15	−81.14
18	−0.10	−0.09	0.00	−94.13
19	−0.40	−0.43	0.04	−101.3
20^ t^	−0.10	−0.07	−0.03	−108.7
21^ t^	−0.18	−0.44	0.27	−101.9
22	0.00	0.00	0.00	−80.97
23	−0.18	−0.15	−0.03	−84.11
24^ t^	−0.18	−0.08	−0.09	−95.84
25	−0.23	−0.21	−0.03	−123.1
26^ t^	−0.06	−0.24	0.18	−101.0
27	−0.48	−0.49	0.01	−105.3
28	−0.74	−0.79	0.05	−117.5
29	−0.78	−0.80	0.02	−110.2
30	−0.74	−0.74	0.00	−80.49
31	0.24	0.20	0.05	−120.4
32^ t^	−0.37	−0.59	0.23	−126.9
33	0.18	0.20	−0.02	−125.1
34	0.11	0.16	−0.04	−118.4
35	−1.16	−1.15	−0.01	−89.72
36	−0.14	−0.14	0.00	−83.71
37	0.17	0.19	−0.01	−95.28
38	−0.92	−0.95	0.03	−88.45
39	0.04	0.01	0.03	−89.74
40	−0.73	−0.72	−0.01	−113.6
41	−1.22	−1.21	−0.01	−130.7
42^ t^	−0.80	−1.15	0.35	−117.9
43	0.00	0.04	−0.04	−90.15
44	0.20	0.20	0.00	−118.4
45	−0.32	−0.33	0.00	−91.68

^t^ Test set.

**Figure 4:**
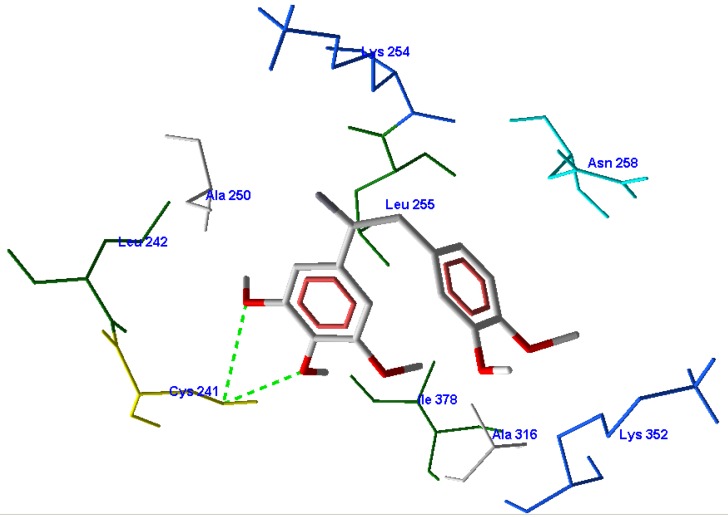
Interactions between the amino acids at colchicine-binding site and the most potent compound 13.

To further study the interaction between inhibitors and protein, the most potent compound 13 was selected to perform the deeper docking study (Figure 4) [39]. 

The *meta*- and *para*-methoxy O atoms of the A-ring can form a hydrogen bond with Cys241. A hydrophobic pocket created by Leu242, Leu255, Ala250 and Val318 was occupied by the trimethoxyphenyl, while the B-ring participated in the hydrophobic effect with Ala316, Lys352, Ile378 and Val315.

### 3.3. CoMFA Results

**Figure 5:**
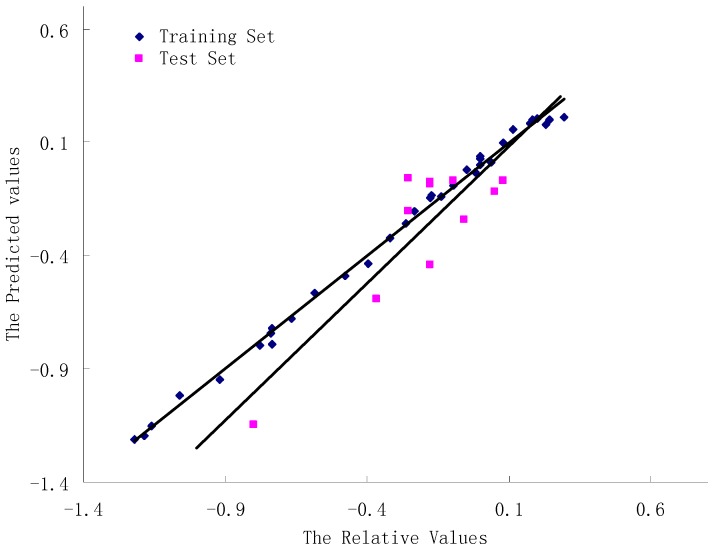
Plot of relative and predicted activities for the training and test set compounds based on the CoMFA model.

The CoMFA study was based on the docking data [40,41]. Alignment was conducted, giving q^2^ value of 0.540. The optimum orientation was derived from AOS, with a satisfactory q^2^ value of 0.786 for five components. Using optimum number of components, the non-cross-validated PLS analysis was conducted to obtain an R^2^ value of 0.988, F value of 472.301 and a standard error of estimated (SEE) of 0.055. The steric and electrostatic field descriptors represent 79.7% and 20.3% dedications, separately. In the Y-randomization validations, the q^2^ values are −0.074, −0.241, −0.188, −0.059, −0.075; and r^2^ values are 0.043, 0.114, 0.117, 0.042, and 0.067. The low q^2^ and r^2^ values show that the good results in our original models are not due to a chance correlation or structural dependency of the training set. All mentioned above suggested that it is feasible to build the 3D-QSAR model through molecular docking alignment. Table 2 shows values with experimental pIC_50_, predicted pIC_50_ and the residues for all compounds. Figure 5 described the relationship between relative pIC_50_ and predicted pIC_50_. The data of CoMFA research are shown in Table 3.

**Table 3:** Summary of the statistical parameters obtained from the CoMFA and analysis.

Statistical parameters CoMFA	CoMFA
the number of training set compounds	34
Components	5
q^2^	0.786
Convention r^2^	0.988
Standard error of estimated	0.055
F values	472.301
Predictive r^2^	0.7412
Fraction	
Steric	79.7%
Electrostatic	20.3%

### 3.4. Validation of the 3D-QSAR Models

If models are reliable toward lead optimization, they must have convincing capability not only in interpolative accurateness but also in extrapolative confirmation [42]. The internal validation of leave-one-out cross validation has been confirmed by the value of q^2^ (0.786). However, as mentioned-above, a high value of q^2^is not the necessary and sufficient condition for a QSAR model to have a better predictive power [43]. Therefore, an external validation was carried out on a set of 11 test compounds not involved in the training set to assess its forecasting ability. As a result, 11 compounds in the test set were all predicted well, with residuals within 1 log unit. Moreover, the CoMFA model gave a good predictive r^2^ value of 0.7412. The test results demonstrate that the CoMFA model we built can be useful in predicting the activities of newly designed inhibitors of tubulin polymerization.

### 3.5. 3D-QSAR Contour Analysis

**Figure 6:**
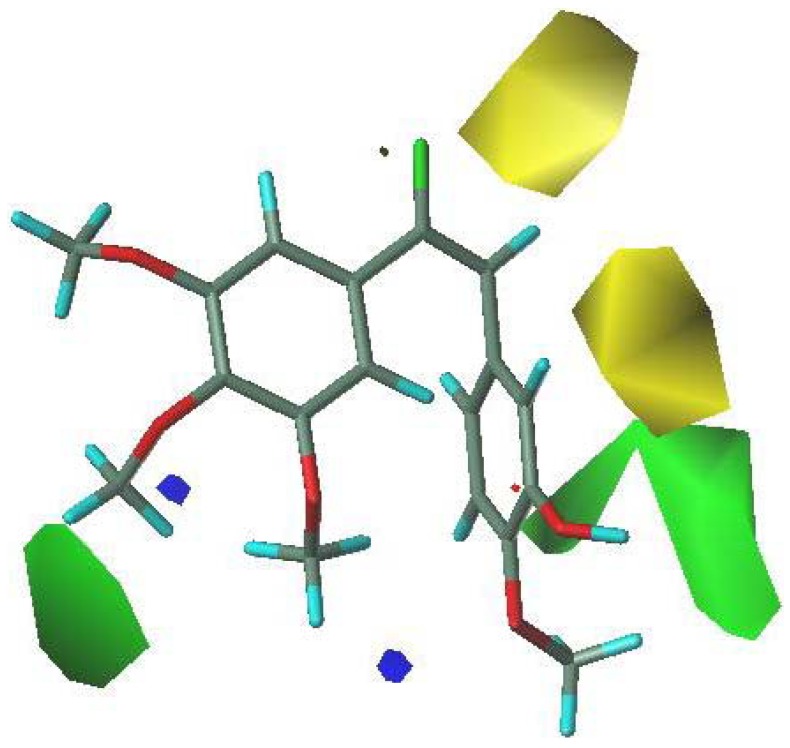
CoMFA STDEV*COEFF contour maps based on compound 12. Green contours emphasize areas that bulky groups are favorable, while yellow contours highlight regions that bulky substituents are unfavorable.Blue contours represent areas where electropositive substituents in these positions will enhance the inhibitory ability on tubulin polymerization while red contours emphasize regions where electronegative groups will increase the inhibitory activity.

The results gained from CoMFA model were graphically converted by means of the stdev*coefficient contour maps (Figure 6) to view the field impact on biological activity. These contour maps offer a more easier understanding of the binding mode of CA-4 analogues and remind us of any change in steric and electrostatic field in three-dimensional space may have effect on biological activity. The contour maps of CoMFA are described with the most potent inhibitors of tubulin assembly (compound 12).

As we can see from the CoMFA contour maps (Figure 6), steric factors influencing biological activity were expressed with green and yellow contour maps. A green region near of the A-ring reveals that bulky groups introduced in these positions are helpful for increasing activity. Compound 8 (0.23) with a thiophene-ring at C-3 and C-4 positions was a potent inhibitor of tubulin polymerization, the biological activity of which was even better than CA-4 (0). When replacing the thiophene-ring with a furan-ring (compound 9), the relative activity was −0.02 and decreased a little, and it was due to that fact the sulfur atom is bigger than an oxygen atom. Another bigger green contour behind the C-3 substituent of the B-ring highlights that larger groups are favorable to promote inhibitory ability, for instance, the biological activity of compound 39 (0.04) was a better than compound 30 (−0.74). Two yellow contours are also shown in Figure 6. One was found above of the C-2 position of the B-ring, the other one was located at some distance from the two-atom chain. These yellow contours indicate that compounds bearing bulky groups in these regions would decrease the ability to inhibit tubulin polymerization.

**Figure 7:**
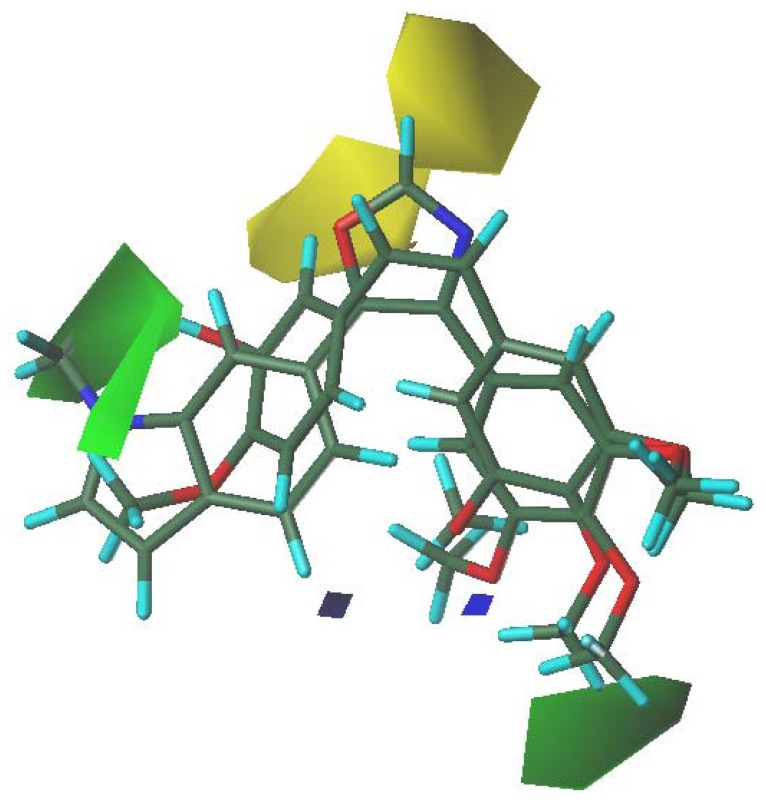
CoMFA STDEV*COEFF contour maps based on compound 33 and CA-4 (1).

For compound 33 (0.18, Figure 7), the oxazole moiety near the yellow contour would decrease the ability to inhibit tubulin polymerization, but the methyl group of the N-methylindole moiety, which inserts into the green area near B-ring, can greatly increase the binding capability. In general, the molecules have the expected activity (0.18). 

The electrostatic contour map shows two small blue contours in Figure 6. These blue contours indicate that compounds bearing electropositive groups, such as compound 15 (0.08), would increase the ability to inhibit the tubulin polymerization.

## 4. Conclusions

A study of molecular docking of CA-4 derivatives linked by a two-atom bridgehead was performed at the colchicine-binding site of tubulin. The binding modes of 45 compounds were identical to that of CA-4, and their interactions with tubulin were evaluated. On the basis of docking alignment, a satisfactory 3D-QSAR model was constructed, whose internal validation can be demonstrated by a q^2^ value of 0.786, high value of R^2^ (0.988), and the external consistency that can be proved by the good predictive R^2^ (0.7412) for the test set. Moreover, the excellent statistical correlations and satisfactory predictive power suggest that it is feasible to build the 3D-QSAR model through molecular docking alignment. In this study, we also concluded: (1) as we can see from the CoMFA contour, the influence of the steric field was more important than that of the electrostatic field; (2) a green contour near the substituent at C-4 of the A-ring reminded us that introducing bulky groups at this position was helpful to enhance biological activity; (3) another bigger green contour behind of the C-3 substituent of the B-ring highlights that larger groups are favorable to promote inhibitory ability; (4) The two yellow contours that one is located above of the C-2 position of the B-ring and another at some distance from the two-atom chain indicate that compounds bearing bulky groups at these regions would decrease the ability to inhibit the tubulin polymerization; and (5) higher electropositive moiety below the *para*-substituent of the A-ring is favorable to enhance the ability to inhibit the tubulin polymerization. 

In summary, the results of the docking study and the analysis of 3D-QSAR provide much useful information for the rational design of novel inhibitors, capable of binding to the colchicine-binding site, displaying selective toxicity towards tumor vasculature and strongly inhibiting tubulin polymerization. Our models would offer help to better comprehend the structure-activity relationships of CA-4 compounds and also provide new solutions for drug design.
